# Hyperactivation of Wnt/β-catenin and Jak/Stat3 pathways in human and zebrafish foetal growth restriction models: Implications for pharmacological rescue

**DOI:** 10.3389/fcell.2022.943127

**Published:** 2022-08-16

**Authors:** Giovanni Risato, Rudy Celeghin, Raquel Brañas Casas, Alberto Dinarello, Alessandro Zuppardo, Andrea Vettori, Kalliopi Pilichou, Gaetano Thiene, Cristina Basso, Francesco Argenton, Silvia Visentin, Erich Cosmi, Natascia Tiso, Giorgia Beffagna

**Affiliations:** ^1^ Department of Biology, University of Padova, Padova, Italy; ^2^ Department of Cardio-Thoraco-Vascular Sciences and Public Health, University of Padova, Padova, Italy; ^3^ Department of Biotechnology, University of Verona, Verona, Italy; ^4^ Department of Women’s and Children’s Health, University of Padova, Padova, Italy

**Keywords:** foetal growth restriction, intrauterine, hypoxia, zebrafish, Wnt, Stat3, pathway, cardiovascular

## Abstract

Foetal Growth Restriction (FGR), previously known as Intrauterine Growth Restriction (IUGR), is an obstetrical condition due to placental insufficiency, affecting yearly about 30 million newborns worldwide. In this work, we aimed to identify and pharmacologically target signalling pathways specifically involved in the FGR condition, focusing on FGR-related cardiovascular phenotypes. The transcriptional profile of human umbilical cords from FGR and control cases was compared with the response to hypoxia of zebrafish (*Danio rerio*) transgenic lines reporting *in vivo* the activity of twelve signalling pathways involved in embryonic development. Wnt/β-catenin and Jak/Stat3 were found as key pathways significantly dysregulated in both human and zebrafish samples. This information was used in a chemical-genetic analysis to test drugs targeting Wnt/β-catenin and Jak/Stat3 pathways to rescue a set of FGR phenotypes, including growth restriction and cardiovascular modifications. Treatments with the Wnt/β-catenin agonist SB216763 successfully rescued body dimensions, cardiac shape, and vessel organization in zebrafish FGR models. Our data support the Wnt/β-catenin pathway as a key FGR marker and a promising target for pharmacological intervention in the FGR condition.

## Introduction

Intrauterine Growth Restriction (IUGR), more recently defined as Foetal Growth Restriction (FGR) (Bendix et al., 2020), refers to a pathological condition in which the foetus fails to grow to its biological potential (weight below the 10th percentile), primarily because of chronic oxygen or nutrient deprivation (placental insufficiency). FGR is an obstetrical condition that affects approximately 10%–15% of pregnant women; in the world, it is observed in about 24% of new-borns and roughly 30 million infants suffer from this condition every year (Suhag and Berghella, 2013; Mu rki and Sharma, 2014). FGR foetuses have approximately a five-to ten-fold increased risk of dying *in utero*, with up to 23%–65% of stillbirths. Therefore, it is important to start early antenatal monitoring to quickly recognize these foetuses and institute timely obstetrical interventions to reduce the perinatal deaths (Suhag and Berghella, 2013).

FGR is known to lead to short-and long-term consequences, in the neonatal period and adulthood, like cardiovascular, respiratory, renal, immunological, neurological and metabolic disease including, but not limited to, hypertension, diabetes, obesity and dyslipidaemia, hence representing a major burden on individuals, society and economy (Bendix et al., 2020).

At present, there is a lack of adequate intrauterine diagnostic approaches and therapeutic interventions in the paediatric period. Thus, dissecting the molecular and cellular mechanisms underlying FGR is a great challenge to improve diagnosis, prognosis and targeted treatment of this clinical entity.

The FGR condition can be determined by different genetic, epigenetic or environmental causes, with foetal or maternal contributions, but the final output is invariably represented by foetal growth restriction due to chronic deprivation of oxygen or nutrients. This is the reason why this condition can be easily modelled in many animal models, either mammalian or non-mammalian, through exposure of the developing organism to hypoxic conditions. So far, FGR has been modelled in several small and large mammals, as well as in chick and zebrafish (*D. rerio*), mostly by inducing hypoxic conditions in the embryo/foetus, thus faithfully mimicking placental insufficiency, without alteration of the maternal health status (Swanson and David, 2015).

The zebrafish embryo develops externally, in a modifiable environment, allowing the real-time visualization of cell signalling communications that are highly conserved in Vertebrates, from fish to humans. Using hypoxic treatments in zebrafish, Kajimura and colleagues successfully identified the Insulin-like Growth Factor Binding Protein 1 (IGFBP1) as an important and targetable player in the hypoxia-induced growth retardation (Kajimura et al., 2005). More recently, Kamei and colleagues provided functional details on how zebrafish IGFBP genes and IGF (Insulin-like Growth Factor) signalling are modulated by hypoxia during embryogenesis (Kamei et al., 2008).

These studies, corroborated by growing evidence on IGF signalling involvement in human FGR (Laviola et al., 2005; Klammt et al., 2008; Martin-Estal et al., 2016), paved the way to the exploitation of zebrafish as a rapid, non-invasive, trustable and screenable model for the identification of key pathways and molecular factors involved in hypoxia-induced FGR, potentially targetable by genetic or pharmacological intervention.

In the last two decades our group has extensively applied zebrafish models to elucidate the role of cell communication during vertebrate development and organ formation by focusing on signalling pathways relevant in embryonic growth; these include Hif/Hypoxia, Jak/Stat3, Wnt/β-catenin, Bmp, Notch, FGF, Hippo/YAP-TAZ, TGFβ, CREB, Shh, Oestrogen and Glucocorticoid signalling (Tiso et al., 2002; Zecchin et al., 2004; Zecchin et al., 2005; Zecchin et al., 2007; Tiso et al., 2009; Moro et al., 2012; Porazzi et al., 2012; Moro et al., 2013; Rampazzo et al., 2013; Benato et al., 2014; Casari et al., 2014; Facchinello et al., 2017; Kim et al., 2017; Vettori et al., 2017; Astone et al., 2018; Giuliodori et al., 2018; Adusumilli et al., 2020; Dinarello et al., 2020; Peron et al., 2020; Facchinello et al., 2021). Many of these genetic studies greatly benefited from the availability of pathway-specific zebrafish lines. These transgenic fish strains allow a fluorescence-based temporal- and spatial-specific analysis of signalling pathway activation *in vivo*, in an intact animal, under selected genetic or environmental conditions, including hypoxia or drug treatments (Corallo et al., 2013; Schiavone et al., 2014; Facchinello et al., 2016).

Taking advantage of these biosensors, we planned to investigate which signalling pathways are either up or downregulated by hypoxia-induced FGR conditions and can potentially act as either adverse or adaptive pathological responses in the developing embryo/larva. In this investigation, we paid specific attention to the zebrafish cardiovascular compartment, one of the most compromised systems in FGR (Visentin et al., 2014; Cohen et al., 2016; Menendez-Castro et al., 2018), histologically comparable with the vascular component of the umbilical cord, a type of sample easily collectable from human FGR and control cases (Peyter et al., 2014).

This is, to our knowledge, the first attempt to perform a wide and comparative multi-pathway screen on human FGR samples and zebrafish models, searching for signalling alterations significantly conserved between these two experimental systems. The final goal is to provide new molecular markers for early diagnosis, and potential targets for pathway-specific treatment of the FGR condition.

## Materials and methods

### Patient recruitment and umbilical cord collection

This study considered 8 women recruited at the University Hospital of Padua (Italy): 4 with AGA (Appropriate for Gestational Age) pregnancy, considered as healthy controls, and 4 with FGR pregnancy. Written informed consent was obtained from each woman before the enrolment, and the protocol (P1826) was approved by the Padua University Hospital Committee for Research on Human Subjects; the study was conformed to the Declaration of Helsinki. Umbilical cord samples were collected and stored in TRIzol reagent (Invitrogen, Life Technologies, Monza, Italy) until total RNA extraction. Sample features are reported in the Results section.

### Maintenance and handling of zebrafish lines

All experiments were performed in accordance with the Italian and European Legislations (Directive 2010/63/EU) and with permission for animal experimentation from the Ethics Committee of the University of Padova (OPBA) and the Italian Ministry of Health (Authorization number 407/2015-PR). *D. rerio* (zebrafish) were kept in a temperature-controlled (28.5°C) environment in a 12:12 light-dark cycle, staged according to Kimmel (Kimmel et al., 1995) and maintained as previously described (Westerfield, 2000).

For anaesthesia or euthanasia of zebrafish embryos and larvae, Tricaine (MS222; E10521, Sigma-Aldrich, Milan, Italy) was added to the fish water at 0.16 mg/ml or 0.3 mg/ml, respectively. The Tricaine overdose for zebrafish euthanasia was performed as recommended by the Zebrafish International Resource Centre (ZIRC, United States: https://zebrafish.org/documents/faq.php). Wild-type lines used in this work included Tuebingen, Giotto and Umbria strains (Pauls et al., 2007).

The following transgenic lines were used: Hif/Hypoxia reporter line *Tg(4xHRE-TATA:EGFP)* ^*ia21*^ (Vettori et al., 2017), Wnt/β-catenin reporter line *Tg(7xTCF-Xla.Siam:GFP)^ia4^* (Moro et al., 2013), Jak/Stat3 reporter line *Tg(7xSRE-HSV.Ul23:EGFP)^ia^* ^*28*^ (Peron et al., 2020), Hippo/YAP-TAZ reporter line *Tg(Hsa.CTGF: EGFP)* ^*ia48*^ (Tiso et al., 2009), TGFβ reporter lines *Tg(12xSBE:EGFP)* ^*ia16*^ (Astone et al., 2018), BMP reporter line *Tg(2xID1BRE:nlsmCherry)* ^*ia17*^ (Collery and Link, 2011), CREB reporter line *Tg(6xCRE:EGFP)* (Facchinello et al., 2017), Notch reporter line *Tg(12xNREbglob:EGFP)* (Parsons et al., 2009), FGF reporter line *Tg(dusp6:d2EGFP)* ^*pt6*^ (Molina et al., 2007), Sonic Hedgehog reporter line *Tg(12xGli-HSVTK:nlsmCherry)* ^*ia10*^ (Dinarello et al., 2020), Glucocorticoid reporter line *Tg(9xGCRE-HSV.Ul23:EGFP)* ^*ia20*^ (Casari et al., 2014), Oestrogen reporter line *Tg(5xERE:GFP)* (Gorelick and Halpern, 2011), thyroid/myocardium transgenic line *Tg(tg:EGFP-myl7:EGFP)* ^*ia300*^ (Facchinello et al., 2017), and vascular/endothelial transgenic line *Tg(fli1:EGFP)* ^*y1*^ (Lawson and Weinstein, 2002).

### Hypoxia treatment

Zebrafish embryos were obtained from adults of the corresponding transgenic lines; transgenic fishes were outcrossed with wild type individuals to ensure fluorescent signal homogeneity in heterozygous offspring. Fluorescent embryos at 24 h post fertilization (hpf) were incubated for 2–5 days at 28°C into a hypoxic chamber (ProOx P110 Oxygen Controller, BioSpherix, Parish, United States) with controlled and constant 5% oxygen concentration (about 2 mg/L, corresponding to a moderate hypoxia). As controls, fluorescent sibs were kept at 28°C outside the hypoxic chamber, under normoxic conditions.

A set of hypoxia treatments was performed following the incubation times applied by Kajimura and colleagues (Kajimura et al., 2005) to re-check the expression of zebrafish *igfbp1a* and *igfbp1b* genes in hypoxic (oxygen at 2 mg/L) and normoxic conditions: 24 h of hypoxia, starting from 1 hpf; 24 h of hypoxia, starting from 24 hpf; 24 h of hypoxia, starting from 48 hpf (Kajimura et al., 2005; Kamei et al., 2008).

### Whole-mount *in situ* hybridization

Antisense riboprobes were synthesized from pME-EGFP and pME-nls-mCherry vectors (Tol2 Kit), after *ApaI* linearization and T7 RNApol transcription. WISH was performed on zebrafish embryos, previously fixed with 4% PFA/PBS and stored in 100% methanol, following standard protocols (Thisse and Thisse, 2008). At least 20 embryos per condition were processed in a single tube. For signal comparison, control and treated embryos were co-processed and Fast Blue co-stained in the same tube; controls were recognized by tail tip excision, performed after PFA-fixation and before WISH. All experiments were performed in triplicate. After WISH, embryos were post-fixed, mounted in 87% glycerol/PBS and imaged in bright field using a dissecting S8APO microscope (Leica, Wetzlar, Germany) equipped with a Digital Sight DS-L3 camera (Nikon, Tokyo, Japan). For confocal imaging of Fast blue-emitted far red fluorescence, embryos were flat-mounted in the same medium and analysed in a DMI6000 inverted microscope with spectral confocal system SP5 (Leica, Wetzlar, Germany).

### RNA isolation and quantitative real time reverse transcription PCR

Total RNA was extracted using the TRIzol Reagent (Invitrogen, Life Technologies, Monza, Italy), following manufacturer’s instructions. Briefly, 100 μg of umbilical cord tissue obtained from human patients and pools of 30 zebrafish embryos for each experimental condition were homogenized in 1 ml of TRIzol Reagent using 5-mm steel beads and 3 cycles of 3 min at 30 mHz in a TissueLyser instrument (Qiagen, Germany). 1 μg of total RNA was used for cDNA synthesis with FIREScript RT cDNA Synthesis KIT (Solis BioDyne, Tartu, Estonia), according to the manufacturer’s protocol. The obtained cDNA was used for gene expression analysis by quantitative real-time PCR (qPCR), focusing on pathway-specific markers (12 pathways considered, plus IGF pathway as control). qPCRs were performed in triplicate with EvaGreen method using 5 x HOT FIREPolEvaGreen qPCR Mix Plus (Solis BioDyne, Tartu, Estonia) and Bio-Rad CFX384 qPCR System (Biorad, California, United States), following the manufacturer’s protocol. All primers were designed using the software Primer3 (http://primer3.ut.ee), with optimal annealing temperature at 60°C. Results were expressed as relative mRNA abundance and normalized to human *GAPDH* or zebrafish *gapdh* as endogenous reference genes. The used primers are reported in Supplementary Table S1.

### Locomotion assay

Behavioural experiments were performed using the DanioVision tracking system (Noldus Information Technology, Wageningen, Netherlands). Zebrafish larvae at 4 dpf (days post-fertilization) were placed in 48-well plates, with one larva per well in 1 ml of fish water. After 20 min of acclimation, movements of larvae were recorded repeating three cycles of 10 min of light and 10 min of dark, for a total duration of 60 min, as previously described (MacPhail et al., 2009).

### Zebrafish length and heart rate measurement

Zebrafish were imaged under a dissecting microscope (Leica M165FC) equipped with a CCD camera (Leica DFC7000T) and controlled by Leica Application Suite (LAS V4.8, Leica Microsystems, Wetzlar, Germany). Embryos at 3 dpf were anesthetized with 0.16 mg/ml Tricaine in fish water to facilitate the body length measurement. Heart rates were determined at 3 dpf by counting the number of atrial contractions during 60-s timeframes. The number of heartbeats per unit of time was expressed as beats per minute (bpm).

### Pharmacological modulation of signalling pathways

Zebrafish embryos, under normoxic or 5% hypoxic conditions, were treated for 48 h with 40 µM SB216763 or 5 µM XAV939 (S3442 and X3004, Sigma-Aldrich, Milan, Italy), directly dissolved in fish water, to activate (SB216763) or inhibit (XAV939) Wnt/β-catenin signalling. Likewise, Jak/Stat3 signalling modulation was carried out by using 50 μM Tyrphostin AG490 (Peron et al., 2020), a known Jak kinase inhibitor, or 20 μM Leukaemia Inhibitory Factor (LIF), a ligand of the Jak/Stat3 pathway that works as agonist (T3434 and H17002, respectively, Sigma-Aldrich, Milan, Italy). Activity of the drugs was checked by treating transgenic reporters for the Wnt/β-catenin and Jak/Stat3 pathways (data available on request).

### Signal quantification and statistical analysis

Signal quantification and morphological analysis were performed using the Measurements option of the Volocity 6.0 software (Perkin Elmer, Milan, Italy). Pairwise analysis was carried out by unpaired *t*-test, while multiple comparisons were analysed by one-way ANOVA followed by Tukey’s test (Graph Pad Prism V7.0 software). In the charts, error bars display standard errors of the mean. The significance threshold for the *p*-value was 0.05. Asterisks indicate significant differences from controls. Correspondence between asterisks and significance levels is indicated in the figure legends.

## Results

### Analysis in foetal growth restriction umbilical cords detects multiple alterations in developmental signalling pathways

To investigate if the FGR condition could induce the modification of a set of signalling pathways, considered for their known relevance in animal development and environmental response, we collected a panel of umbilical cord samples from human FGR foetuses and healthy (AGA) controls (Table 1).

**TABLE 1:** Collection of FGR and AGA umbilical cord samples. AGA: Appropriate for Gestational Age, FGR: Foetal Growth Restriction.The Apgar score is used to quantify the neonatal wellbeing, based on five parameters (Appearance, Pulse, Grimace, Activity, Respiratory effort). Scores of seven or above are considered normal.

Sample	Diagnosis	Mother’s age (years)	Gestation week	Weight (grams)	Sex	Length (cm)	Percentile	Apgar
1	AGA	29	36	3,180	F	49	80	9
2	AGA	32	38	2,920	M	47	47	9
3	AGA	35	39	3,580	M	49	52	9
4	AGA	30	39	3,430	F	50	50	9
5	FGR	40	39	2,095	F	45	<3	9
6	FGR	29	38	2,100	F	43	<3	9
7	FGR	33	40	2,730	M	47	5	9
8	FGR	41	38	2,220	F	46	<3	9

**FIGURE 1:**
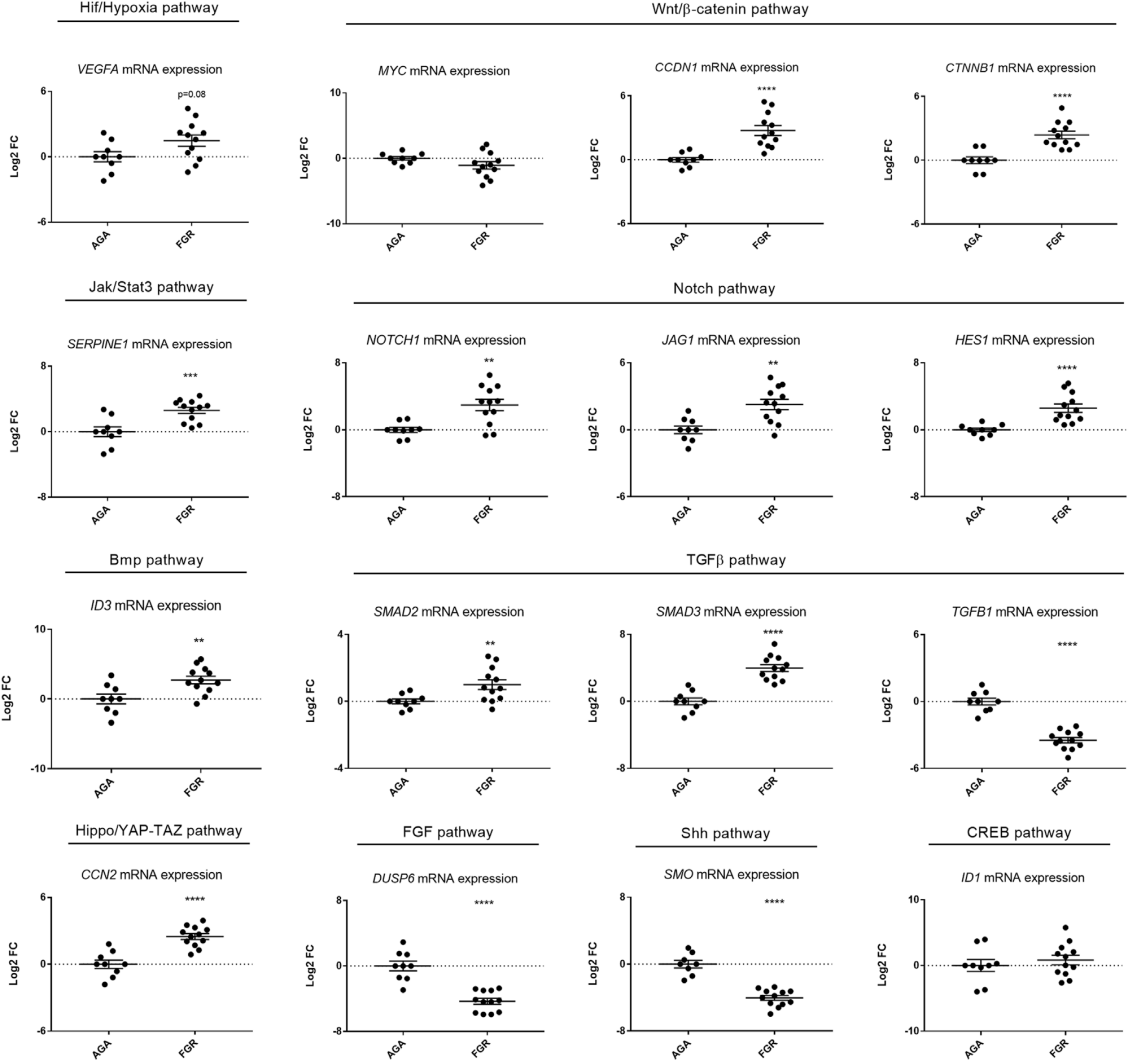
Signalling pathway dysregulation in human FGR samples. Quantitative RT-PCR analysis performed on umbilical cords from FGR cases and AGA controls, focusing on ten signalling pathways, identified significant upregulation in seven pathways (Hif/Hypoxia, Wnt/β-catenin, Jak/Stat3, Notch, Bmp, TGFβ, Hippo/YAP-TAZ), downregulation in two (FGF, Shh) and no modification in one (CREB). Sample size: *n* = 8. * = *p* < 0.05; ** = *p* < 0.01; *** = *p* < 0.001; **** = *p* < 0.0001, Log2 FC stands for Log2 Fold Change. Test: Unpaired t-test.

Both FGR and AGA samples were analysed by quantitative real-time PCR, focusing on members of the following pathways: Jak/Stat3, Wnt/β-catenin, Bmp, Notch, FGF, Hippo/YAP-TAZ, TGFβ, CREB, Shh, Oestrogen and Glucocorticoid, including IGF and Hif/Hypoxia as controls to validate the experimental system in terms of hypoxia response and comparability with the zebrafish model. The IGF member *IGFBP1* showed significant downregulation, while the *IGFBP7* gene was slightly upregulated (*p* = 0.07) (Supplementary Figure S1), suggesting differential regulation of the IGF pathway between FGR and AGA cases. Concerning the Hif/Hypoxia signalling (shown to be upregulated in hypoxia-treated zebrafish (Santhakumar et al., 2012), we detected a slight upregulation of the pathway in human FGR samples, as deduced from the human target gene *VEGFA* (*p* = 0.08) (Figure 1).

Overall, both control pathways, IGF and Hif/Hypoxia signalling, showed differential activity in human FGR samples when compared to normal (AGA) controls, similarly to what already demonstrated in hypoxia-induced zebrafish FGR models. We did not detect any significant change in the expression of pathway members for Oestrogens and Glucocorticoids (not shown) as well as for CREB (Figure 1), when comparing AGA and FGR umbilical cordons; hence, these pathways were not considered for further analysis. Conversely, significant changes were detected in all other signalling pathways (Figure 1). Specifically, Wnt/β-catenin (2 out of 3 markers), Jak/Stat3, Bmp, Notch (3 out of 3 markers), TGFβ (2 out of 3 markers) and Hippo/YAP-TAZ resulted upregulated, while Shh and FGF were downregulated in FGR cases, compared to AGA controls (Figure 1). A summary of the pathway screen is shown in Table 2.

**TABLE 2:** Summary of the signalling pathway screening in human and zebrafish FGR samples. Pathways are grouped, from top down, as: upregulated in both species, differentially regulated in both species; undetectable or unchanged in both species; downregulated in both species. Significantly upregulated pathways are indicated by orange colour and up arrow(s). Significantly downregulated pathways are indicated by green colour and down arrow(s). Unchanged pathways are indicated by yellow colour and horizontal arrows. Multiple arrows indicate multiple markers tested. Smaller arrows and lighter colour indicate trends (the p value is included). Not detected markers are indicated by n.d. and white background.

Signalling pathway	Human umbilical cord	Zebrafish vessels	Zebrafish heart	Zebrafish brain
Hif/Hypoxia	↑ (*p* = 0.08)	↑	↑	↑
Wnt/β-catenin	↑↑↑↓	↑	↑	↑
Jak/Stat3	↑	↑	↑	↑
Bmp	↑	↑	←→	←→
Notch	↑↑↑	←→	←→	←→
Hippo/YAP-TAZ	↑	←→	↓	←→
TGFβ	↓↑↑	←→	↓	↓
Shh	↓	←→	↓	↑
FGF	↓	←→	←→	↑
CREB	←→	←→	←→	←→
Oestrogen	n.d	←→	←→	←→
Glucocorticoid	n.d	←→	←→	↓
	Hum. umb. cord	Whole zebrafish embryo		
IGF	↓ ↑ (*p* = 0.07)	↓		

### Both short-and long-term hypoxia leads to IGF signalling changes in zebrafish embryos

The pathway screen on human samples gave us strong evidence of signalling dysregulation and/or adaptive stress response in FGR foetuses and prompted us to verify if these variations could be recapitulated in a hypoxia-induced zebrafish model for the FGR condition. Indeed, such comparison could provide some indications on which mechanisms are more robustly conserved between the two species, and globally modified when animal growth is restricted.

As a first step for FGR modelling in zebrafish, we verified, in our system, if hypoxia-treated zebrafish embryos could display IGF signalling dysregulation. Specifically, we found that, after 24 h-treatments with 5% hypoxia, the levels of *igfbp1a* were significantly upregulated in 1 and 2 dpf zebrafish embryos, with subsequent decrease at 3 dpf, while for the other paralog *igfbp1b* the expression was initially decreased (at 1 dpf), followed by a slight increase (Supplementary Figure S2A). Overall, after 1-day treatments with hypoxia, both genes displayed expressional dynamics comparable with those described by Kamei and colleagues (2008).

While these preliminary checks support data reproducibility when measuring dynamic changes of gene expression in zebrafish FGR models, we wanted to further investigate how gene expression could be modified after a long-term hypoxia exposure. In principle, a continuous hypoxic condition should more faithfully mimic chronic oxygen deprivation during development, allowing to detect gene expression levels resulting from a sustained modification of signalling pathway activity. To this purpose, we first allowed zebrafish embryos to reach the 1-dpf stage, without interfering with early embryonic development (cleavage, blastulation and gastrulation), also considering that this developmental time corresponds to human stages likely unaffected by placental insufficiency (the umbilical cord forms at later times, around week 3). We incubated zebrafish embryos in 5% hypoxia from 1 dpf and analysed gene expression at 3 dpf. When re-evaluating *igfbp* expression after this treatment protocol, we found that *igfbp1a* was substantially unaffected, while *igfbp1b* was strongly downregulated (Supplementary Figure S2B), suggesting that temporal modification of hypoxia exposure could still induce IGF signalling adjustments, but with different outputs in terms of gene expression levels, likely reflecting long-term adaptations of the developing embryo to chronic hypoxia exposure.

### Long-term hypoxia treatment induces growth restriction in zebrafish embryos

**FIGURE 2:**
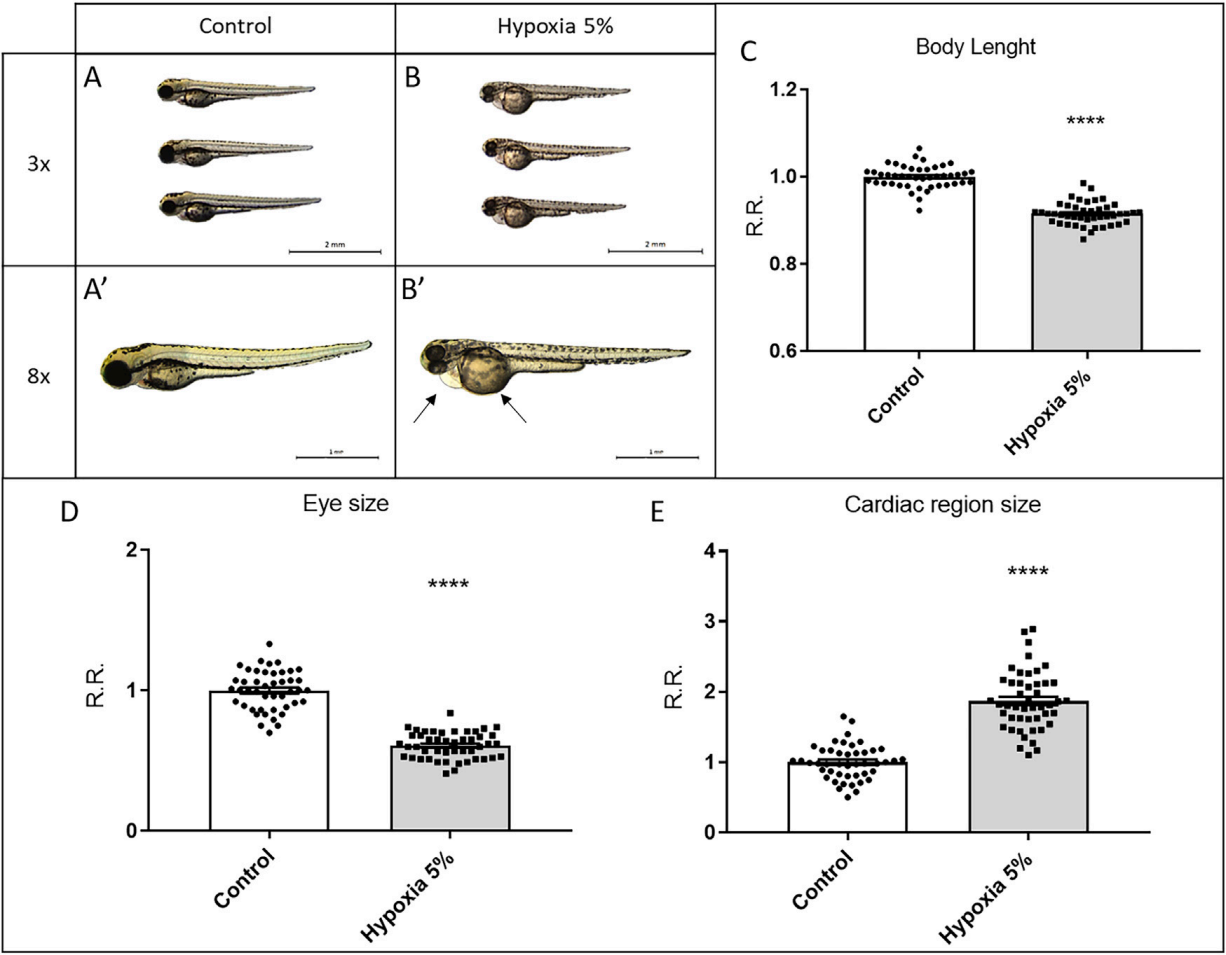
Long-term hypoxia treatment leads to growth restriction in zebrafish embryos. **(A–E)** Morphological analysis of 3-dpf zebrafish embryos after a long-term (2-days long) treatment in 5% hypoxia; hypoxia-treated embryos display reduced body length **(A–C)** and ocular size **(D)**, poorly absorbed yolk and a pericardial effusion (arrows in B′) leading to a significant enlargement of the cardiac region size **(E)**. All embryos are in lateral view, anterior to the left. Sample size: *n* = 45; **** = *p* < 0.0001; R.R. = Relative Ratio. Test: Unpaired t-test.

After applying our protocol of long-term hypoxia, we morphologically inspected the treated embryos, observing growth restriction. Hypoxia-incubated embryos displayed, all at statistically significant levels, decrease of the body length, non-isometric reduction of the ocular region, and lack of yolk absorption (Figure 2), all of them fundamental parameters to determine the developmental stage in 1–3 dpf zebrafish embryos (Kimmel et al., 1995). Based on these data, 3-dpf embryos in the hypoxia group were developmentally retarded, being morphologically equivalent to control embryos at 2 dpf. Moreover, hypoxia-treated embryos displayed a peculiar cardiac phenotype, characterized by pericardial effusion (Figure 2), although in the absence of significant heart rhythm alteration (not shown).

### Prolonged hypoxia induces cardiovascular modifications in zebrafish foetal growth restriction models

**FIGURE 3:**
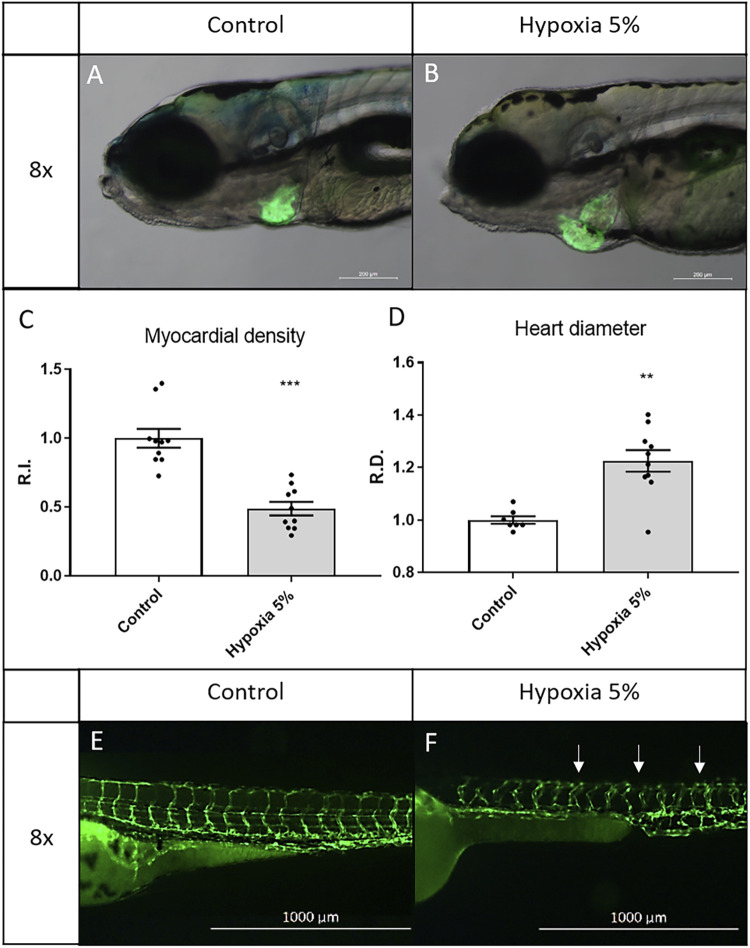
Long-term hypoxia induces cardiovascular modifications in zebrafish embryos/larvae. **(A–D)** After a 5-days incubation in 5% hypoxia, 6-dpf zebrafish larvae display enlarged heart and inflated myocardial layer, as evidenced by the fluorescence of the myocardial transgene *Tg(tg:EGFP-myl7:EGFP)^ia300^*. **(E,F)** After a 2-days incubation in 5% hypoxia, 3-dpf zebrafish embryos display disorganized intersegmental vessels (arrows), identified by the endothelial transgene *Tg(fli1:EGFP)^y1^*. 6-dpf larvae and 3-dpf embryos are in lateral view, anterior to the left. Sample size: *n* = 10; ** = *p* < 0.01; *** = *p* < 0.001; R.I. = Relative Intensity; R.D. = Relative Diameter. Test: Unpaired t-test.

The pericardial effusion, observed in hypoxia-treated embryos, prompted us to perform a more in-depth analysis of the cardiovascular modifications under chronic oxygen deprivation, exploiting heart- and vessel-specific transgenes and 5-days long incubations in hypoxic conditions. After 5 days under 5% hypoxia starting from 1 dpf, zebrafish larvae were inspected at the cardiac level, using the myocardial-specific transgene *Tg(tg:EGFP-myl7:EGFP)* ^*ia300*^. By analysing its fluorescent pattern at 6 dpf, we could detect a substantial enlargement of the heart (22%) and, as deduced from the rough halving of the fluorescence after hypoxia treatment, a significant thinning of the expanded myocardial layer in the heart chambers (atrium and ventricle) (Figure 3). For the morphological analysis of the vascular tree, we took advantage of the endothelial line *Tg(fli1:EGFP)^y1^*, where we observed a global disorganization, both in orientation and ramification, of intersomitic vessels in hypoxia-treated larvae, compared to untreated siblings (Figure 3). Overall, heart and vessel modifications indicate strong impairment and/or long-term adaptation of the embryonic cardiovascular system following prolonged hypoxia, representing easily examinable markers to assess the effects of potential therapies in FGR modelling.

### Long-term hypoxia affects zebrafish embryo locomotion

To complete the morpho-functional characterization of the zebrafish FGR models, we took into consideration their locomotor performances as well. After a 2-days incubation in 5% hypoxia, 4-dpf larvae were imaged for 1 h under alternating light/dark periods. This test allowed to detect a normal response to light stimuli in hypoxia-treated larvae, but with a significant reduction of the total distance swum, compared to untreated siblings (Supplementary Figure S3), suggesting preserved sensory perception, but impaired motor response after hypoxia treatment.

### Long-term hypoxia modifies multiple pathways in the zebrafish cardiovascular and brain regions

The morpho-functional characterization of zebrafish embryos under long-term hypoxia allowed us to confirm them as *bona fide* models of FGR condition, in terms of IGF signalling dysregulation and delayed/impaired development of anatomical and locomotor features. We thus started a large-scale screen of developmental pathways, taking advantage of a wide collection of zebrafish biosensors, available at the UniPD Zebrafish Centre, able to report *in vivo*, though fluorescent reporters, the activation of cell signalling cascades (Corallo et al., 2013).

While the zebrafish translucency allows to directly quantify the reporter protein levels from their emitted fluorescence, we nonetheless opted for a post mortem analysis of the reporter mRNA, which is more dynamically produced and degraded, and thus more faithfully informative on the real timing of up/down regulation of a given pathway (Corallo et al., 2013).

**FIGURE 4:**
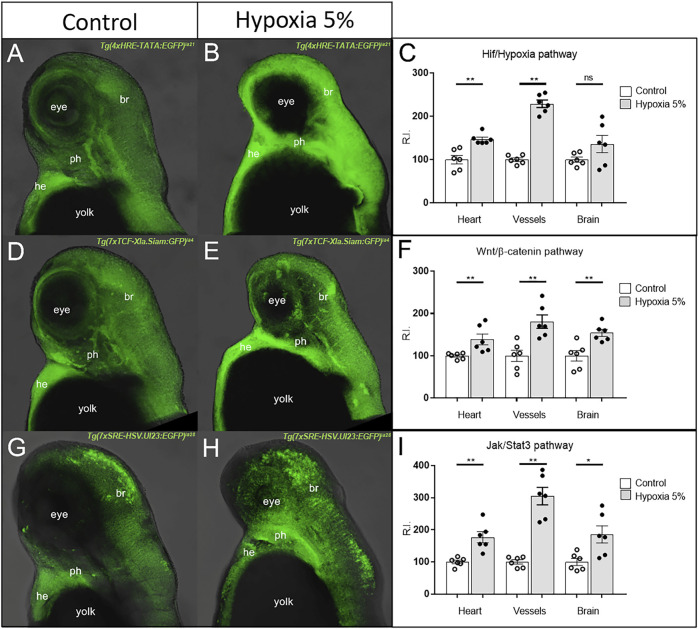
Hyper-activation of Hif/Hypoxia, Wnt/β-catenin and Jak/Stat3 signalling in the cardiovascular and brain regions of hypoxia-treated zebrafish. The GFP-based (green) reporters for Hif/Hypoxia **(A–C)**, Wnt/β-catenin **(D–F)** and Jak/Stat3 **(G–I)** signalling show upregulation of the three pathways in all considered anatomical regions. All embryos are at 3 dpf and displayed in lateral view, anterior to the top. br = brain; he = heart; ph = pharynx. Sample size: *n* = 6 per condition; ns = not significant; * = *p* < 0.05; ** = *p* < 0.01; R.I. = Relative Intensity. Test: Unpaired t-test.

Specifically, 1 dpf zebrafish embryos from 12 considered transgenic lines (including Hif/Hypoxia reporter as internal control) were maintained for 2 days under normoxia or 5% hypoxia, then euthanized and fixed at 3 dpf; finally, each pathway reporter expression was detected by fluorescent WISH and confocal-imaged. We focused our attention on heart and pharyngeal vessels, due to the heavy involvement of the cardiovascular system in FGR; as additional anatomical district, we took into consideration the forebrain region. The Hif/Hypoxia reporter invariably confirmed the upregulation of the pathway in hypoxia-treated embryos; moreover, Wnt/β-catenin and Jak/Stat3 signalling (both already reported to be master regulators of Hypoxia response) (Xu et al., 2017; Dinarello et al., 20212021) were also strongly upregulated in all anatomical regions inspected (Figure 4). TGFβ signalling was downregulated in all considered regions, while the remaining pathways were unmodified or mildly affected in a subset of the considered tissues (Supplementary Figures S4, S5). Comparing these results with data obtained from human samples, we noticed that in both species Wnt/β-catenin and Jak/Stat3 signals were strongly affected under FGR condition, and thus worth to be investigated on their role as hypoxia-induced pathogenic or adaptive mechanisms.

### Drug-mediated modulation of Wnt/β-catenin, but not Jak/Stat3, can rescue body length and pericardial morphology in foetal growth restriction models

**FIGURE 5:**
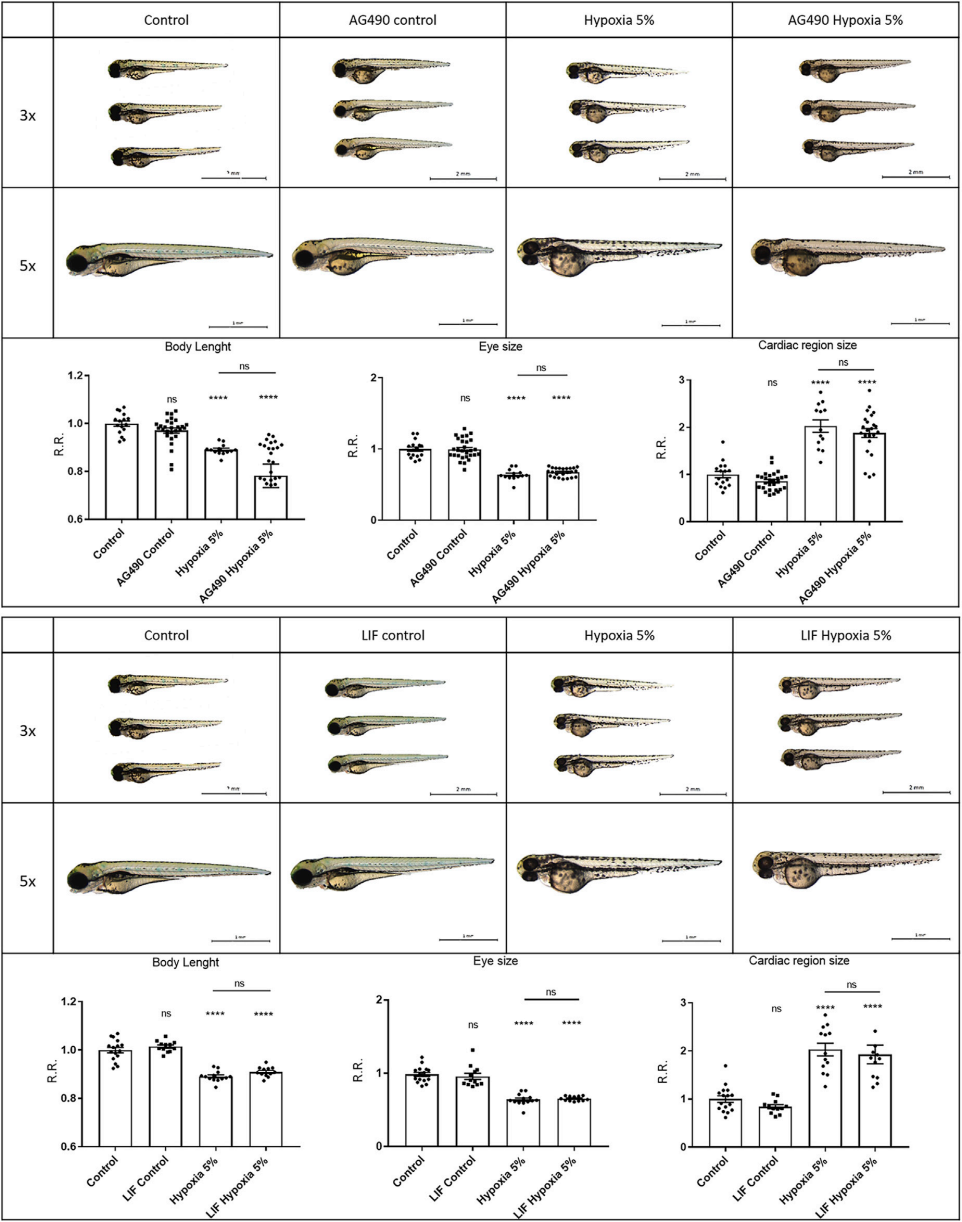
Stat3 signalling modulation does not rescue FGR anomalies. Downregulation of Stat3 signalling with 50 μM AG490 inhibitor does not rescue body length, eye size and cardiac morphology under hypoxia. Upregulation of Stat3 signalling with 20 μM LIF agonist does not rescue body length, eye size and cardiac morphology under hypoxia. All embryos, at 3 dpf, are displayed in lateral view, anterior to the left. Sample size: *n* = 15; **** = *p* < 0.0001; ns = not significant; R.R. = Relative Ratio. Test: One-way ANOVA followed by Tukey’s test.

**FIGURE 6:**
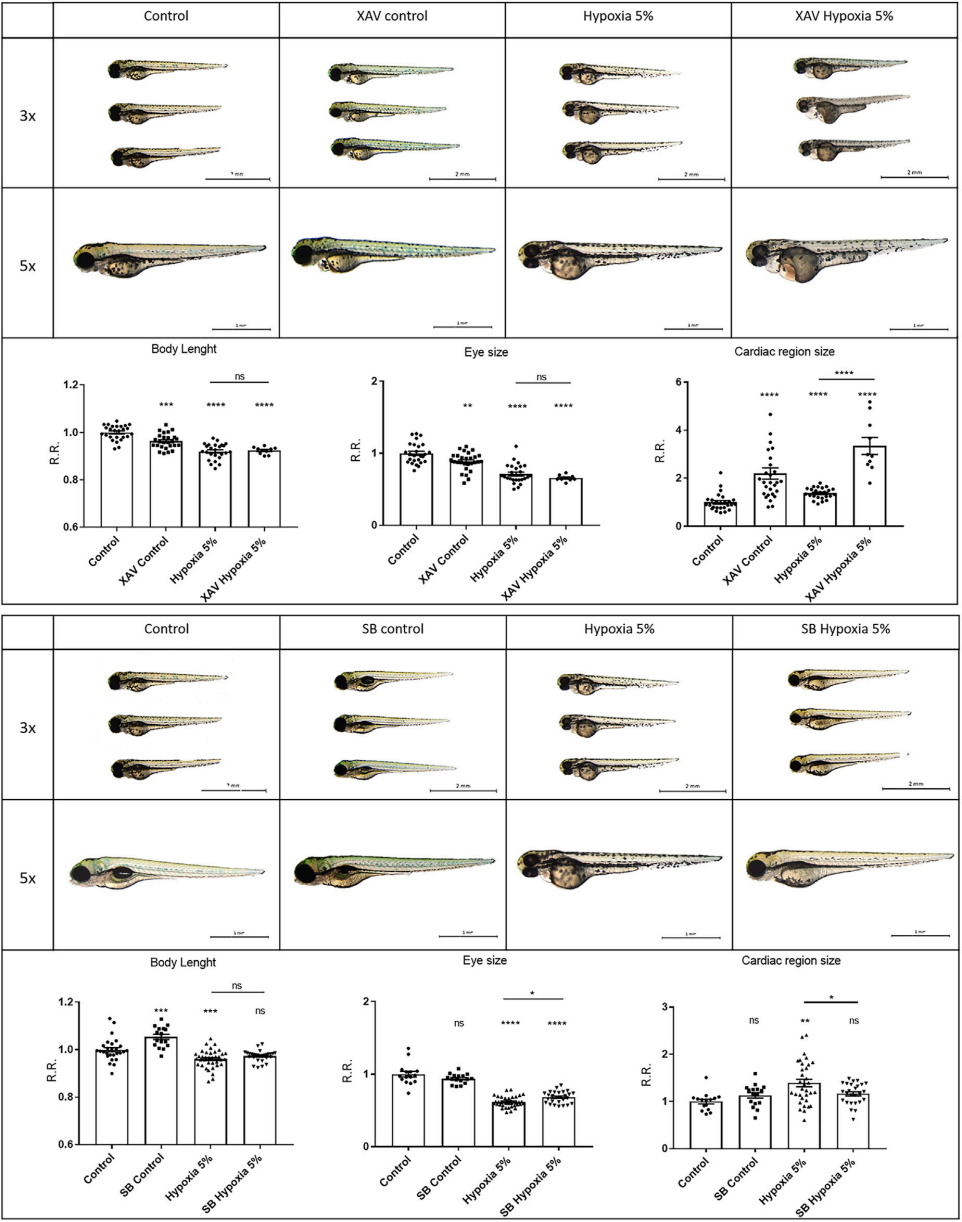
Wnt/β-catenin signalling upregulation can rescue FGR anomalies. Downregulation of Wnt/β-catenin signalling with 5 µM XAV939 (XAV) inhibitor does not rescue body length or eye size, and worsens the pericardial effusion under hypoxia. Upregulation of Wnt/β-catenin signalling with 40 µM SB216763 (SB) agonist can rescue the body length and the pericardial effusion under hypoxia. All embryos, at 3 dpf, are displayed in lateral view, anterior to the left. For display purposes, images of control embryos under normoxia and hypoxia correspond to control images of Figure 5, as the same control conditions were used for both Stat3 and Wnt experiments. Sample size: *n* = 15; * = *p* < 0.05; ** = *p* < 0.01; *** = *p* < 0.001; **** = *p* < 0.0001; ns = not significant; R.R. = Relative Ratio. Test: One-way ANOVA followed by Tukey’s test.

To elucidate the potential significance of Wnt/β-catenin and Jak/Stat3 signals as therapeutic targets in FGR, we simultaneously exposed zebrafish embryos to hypoxic conditions (2-days incubation) and specific agonists/inhibitors targeting these pathways, to analyse their combined effects on FGR phenotypes. For the Jak/Stat3 signalling pathway, we exposed both normoxia- and hypoxia-treated embryos to either the Stat3 inhibitor AG490 or the Stat3 agonist LIF. Neither the Stat3 inhibition by AG490 nor the Stat3 upregulation by LIF had significant effects in rescuing body length, eye size or pericardial effusion under hypoxia (Figure 5). This suggests that Stat3 signalling, although modified and possibly involved in other adverse or adaptive functions, is not playing any major role in the causation of the observed FGR-like anomalies. Concerning the Wnt/β-catenin signalling, we downregulated the pathway with the inhibitor XAV939: this treatment had no rescuing effect neither in the body length nor in the eye size, and worsened the pericardial effusion under hypoxia. Conversely, the upregulation of Wnt/β-catenin signalling with the agonist SB216763 displayed rescuing effects on FGR phenotypes, by increasing the body length and reducing the pericardial effusion (Figure 6), suggesting a key role for Wnt/β-catenin as a major adaptive pathway whose activation counteracts hypoxia-induced effects on body growth and pericardial homeostasis.

### Wnt/β-catenin upregulation can rescue hypoxia-induced vascular defects

**FIGURE 7:**
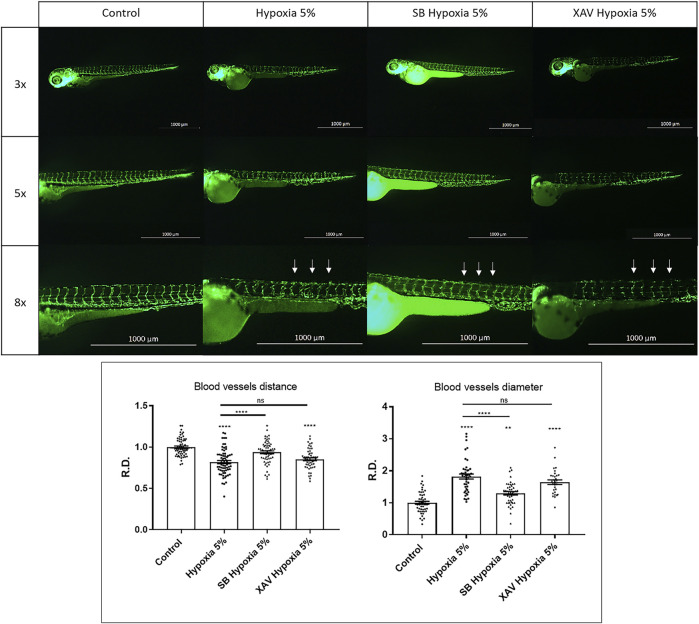
Wnt/β-catenin upregulation can rescue hypoxia-induced vascular defects. Upregulation of Wnt/β-catenin signalling using 40 µM SB216763 (SB) can rescue the vascular organization (reciprocal distance and vessel diameter) in hypoxia-treated embryos. Conversely, downregulation of Wnt/β-catenin signalling using 5 µM XAV939 leads to a worsened phenotype. Intersegmental vessels (arrows) were inspected in 3 dpf embryos, after 2-days long incubation in normoxia (control) or 5% hypoxia, exploiting the endothelial-specific transgene *Tg(fli1:EGFP)^y1^*. All pictures display trunk regions in lateral view, anterior to the left. Sample size: *n* = 15; ns = not significant; * = *p* < 0.05; ** = *p* < 0.01; **** = *p* < 0.0001; R.D. = Relative Distance. Test: One-way ANOVA followed by Tukey’s test.

The remarkable effects of Wnt/β-catenin upregulation in rescuing FGR anomalies prompted us to further investigate its role at the cardiovascular level. To this purpose, we compared the activity of the Wnt/β-catenin modulators SB216763 and XAV939 on the organization of the vascular tree under hypoxia. After treatment with the Wnt/β-catenin agonist SB216763, performed on the vessel-specific transgenic line *Tg(fli1:EGFP)* ^*y1*^, we could observe a rescue of the intersomitic vessels organization, ramification, reciprocal distance and diameter. Conversely, the Wnt/β-catenin inhibitor XAV939 led to an exacerbation of the vascular disorganization, further corroborating the notion that Wnt/β-catenin upregulation may represent a mechanism counteracting hypoxia-induced vascular modifications (Figure 7). Finally, to check if the rescuing effects of the Wnt/β-catenin pathway might occur *via* IGF signalling modulation, we analysed *igfbp1a*/*igfbp1b* expression under hypoxia and agonist-mediated Wnt/β-catenin hyper-activation. This set of experiments showed that the chemical upregulation of Wnt/β-catenin signalling under hypoxia can upregulate *igfbp1a* and rescue *igfbp1b* levels (Supplementary Figure S2C). This suggests that the therapeutic effects of the Wnt/β-catenin pathway on FGR phenotypes may be exerted, at least in part, by IGF signalling rescue. A model of Wnt/IGF signalling interaction is shown in Supplementary Figure S2D.

## Discussion

In this work, we have exploited a hypoxia-induced zebrafish model of FGR for a large-scale screen of signalling pathways, relevant in animal development, in comparison with a panel of human samples, represented by umbilical cords from FGR foetuses and AGA controls. The final goal was the identification of key mechanisms with relevance to short- or long-term FGR pathogenesis, potentially reflecting foetal adaptations to a limited environment, especially at the metabolic and cardiovascular level, in line with the so-called Barker hypothesis of the “thrifty phenotype” (Barker, 2006; Cosmi et al., 2009; Cosmi et al., 2011).

### Induction of multiple foetal growth restriction-like phenotypes in zebrafish after prolonged hypoxia

As a first step in refining a zebrafish-based modelling of FGR, our study has demonstrated that prolonged exposure of zebrafish embryos to 5% hypoxia is a suitable protocol to re-identify the IGF signalling as a key molecular signature in individuals with growth restriction. Moreover, the hypoxia-treated zebrafish faithfully mimic a set of morphological and functional features detected in FGR cases. Specifically, our zebrafish models invariably present with growth retardation and delay in the nutrient uptake, as deduced from the reduced body length and the underused yolk reserves.

The pericardial effusion is another feature consistently detected in our models. This aspect is particularly relevant, considering retrospective reviews of prenatally diagnosed pericardial effusions, where this clinical sign has been associated with FGR (Kyeong et al., 2014).

Moreover, we have shown that zebrafish FGR models are characterized by a disorganized vascular tree, remarkably in line with the altered vascular architecture detected in FGR foetuses and umbilical cords (Cosmi et al., 2009; Society for Maternal-Fetal Medicine Publications et al., 2012) and with the impaired vascular growth found to persist in adolescents who suffered FGR (Brodszki et al., 2005).

Finally, our FGR zebrafish display impaired movements. Being the dark/light response still preserved in our models, this would suggest a specific motor defect. These results are particularly relevant in supporting the zebrafish as an excellent model to study, at embryonic and larval stages, early signs of FGR impairments, since locomotor deficiencies have been indeed described in FGR human infants (Padilla et al., 2011), as well as in mammalian models for FGR at the neonatal and adult stage, including mice (Vucetic et al., 2010), rabbits (Lopez-Tello et al., 2019) and rats (Rains et al., 2021).

### Identification of common pathways in human and zebrafish foetal growth restriction models

As initially anticipated, the most salient results of this study have been obtained from the screen of signalling pathways, both in human samples and zebrafish embryos. This is, so far, the first large-scale screen of signalling pathways performed *in vivo*, in a whole vertebrate animal, aimed at modelling the FGR condition. The study has led to the identification of a panel of signals differently expressed in FGR and control individuals, in some cases with striking similarities between the two species (overview available in Table 2).

The IGF and Hif/Hypoxia pathways, considered as internal controls due to their known involvement in FGR (IGF) and hypoxia response (Hif/Hypoxia signalling), confirmed their differential expression in both species, supporting the reliability of this experimental setup.

A subset of pathways, represented by CREB, Oestrogen and Glucocorticoid signalling, were undetectable or unmodified in samples from both species. This was an unexpected result as steroid hormone activities, by the way strongly interlaced with CREB signalling (Jing et al., 2016) and hypoxic response (Vettori et al., 2017; Marchi et al., 2020) have been previously linked to FGR (Solano and Arck, 2019). On the other hand, our observations were limited to data from zebrafish embryonic stages and whole umbilical cords (and not, for instance, from blood-enriched samples); thus, a possible involvement of these endocrine signals in other contexts, such as different tissues or developmental stages, shall not be excluded.

The remaining pathways, representing half of the considered collection, displayed significant changes between FGR and control cases. A set of pathways (Bmp, Notch, Hippo/YAP-TAZ, TGFβ, Shh, and FGF) were variably modified not only based on FGR versus control comparisons, but also depending on the species and tissue types, suggesting a complex and dynamic regulation of these signals, with organ-or organism-specific up/down-regulation. For most of these pathways the link with FGR has not been fully clarified before. Bmp and TGFβ signalling have been analysed in post-natal FGR lung as fibrosis markers (Alcazar et al., 2014); moreover, together with Notch signalling, Bmp and TGFβ have been found deregulated in relation to embryonic vascular malformations (Cunha et al., 2017). Interestingly, also the Hippo/YAP-TAZ signalling has emerged as a key pathway in vascular development (Park and Kwon, 2018), while a pro-angiogenic role during embryonic/foetal development has been long recognized for FGF and Shh (Barut et al., 2010; Chapouly et al., 2019). It is thus tempting to speculate that the dysregulation of this group of pathways in human samples may reflect pathological changes or physiological adjustments specifically occurring in the vascular component of the umbilical cord under FGR condition. Although this group of pathways is certainly worth to be further investigated, especially through targeted modulation in a model system, we gave priority to another set of pathways, represented by Wnt/β-catenin and Jak/Stat3, found consistently upregulated in human FGR samples as well as in hypoxia-treated zebrafish embryos. Notably, in zebrafish these pathways appeared hyper-activated in all considered anatomical districts (vessels, heart and brain), suggesting a strong involvement of these signalling cascades in a generalized pathogenetic mechanism and/or physiological adaptation of the whole organism under hypoxia-induced FGR condition. On the other hand, the hypoxic environment is a known inducer of epithelial-to-mesenchymal transition (EMT) during embryonic development, involving known EMT-triggering pathways such as Wnt/β-catenin and Jak/Stat3, as well as TGFβ and Notch signalling (all upregulated in our collection of human samples) (Cui et al., 2016).

Therefore, we have verified whether the pharmacological up- or down-regulation of Wnt/β-catenin or Jak/Stat3 signals might rescue or further exacerbate body growth indicators and cardio-vascular parameters in our zebrafish FGR models.

### Pharmacological modulation of Jak/Stat3 signalling in zebrafish foetal growth restriction models

The chemical modulation of Jak/Stat3 signalling could not significantly modify the hypoxia-induced morphological phenotypes, suggesting a limited role of this pathway on body growth and pericardial homeostasis. However, an involvement of Jak/Stat3 on other physiological parameters has not been investigated and thus could not be excluded; this is especially relevant considering that decreased STAT3 expression in placenta has been proposed to contribute to abnormal trophoblast function in idiopathic FGR-affected pregnancies (Borg et al., 2015). Also, Jak/Stat3 has a known cardioprotective effect, through both genomic functions as a transcriptional factor—e.g., in angiogenesis (Chen and Han, 2008) and by playing non-genomic roles as a regulator of autophagy and hypoxia-induced ROS in mitochondria (Harhous et al., 2019). For all these aspects and considering the significant dysregulation of Jak/Stat3 signalling as detected in our study, this pathway still represents a very attractive target for therapeutic intervention in FGR.

### Pharmacological rescue of zebrafish foetal growth restriction phenotypes by Wnt/β-catenin-directed drug treatment

Concerning the Wnt/β-catenin signalling, the up- or down-regulation of this pathway in zebrafish could instead significantly affect body growth, pericardial size and vascular morphology under hypoxia, leading to an ameliorated or worsened phenotype in case of agonist or inhibitor treatment, respectively. Of note, a preliminary analysis of IGF signalling members *igfbp1a*/*igfbp1b* indicates that the rescuing effects of the Wnt/β-catenin pathway may be exerted, at least in part, by IGF signalling modulation. These findings suggest that sustaining an already hyper-activated Wnt/β-catenin signalling in FGR may represent a rational intervention to support body growth and vascular development, while counteracting cardiac alterations such as the pericardial effusion.

Notably, the Wnt/β-catenin agonist used in this study-(SB216763)—is a drug already applied in human therapy, especially at the endothelial level, proven to reduce infarct size and prevent cardiac ischemia (Obame et al., 2008; Valerio et al., 2011). Further investigations, exploiting the zebrafish models, are required to clarify if targeting Wnt/β-catenin in FGR could be an effective strategy using short-or, rather, long-term treatments, if toxic or side effects can be induced in the cardiovascular system or in other anatomical districts, and if the therapeutic effects can be maintained also at juvenile and adult stages. Also, current limitations of this study, related to the strict focus on the cardiovascular system and the zebrafish embryonic stage, could be overcome by re-checking signal cross-talks and pathway-directed drugs on an expanded collection of human FGR samples and zebrafish models analysed in other anatomical districts and at different time points. If, under a varying context, the considered pathways could confirm the significant expressional changes here identified, and the extraordinary rescuing effects observed for the Wnt/β-catenin signalling, they would represent easily detectable, robust and promising markers for FGR diagnosis, prognosis and treatment.

### Conclusions and translation perspectives

The discovery of the involvement of the Wnt/β-catenin pathway as a signal differentially dysregulated in both human and zebrafish FGR models points to this pathway as a robust marker for Foetal Growth Restriction. Moreover, the successful rescue of FGR-related phenotypes such as body dimension, cardiac shape and vessel organization, mediated by the Wnt/β-catenin agonist SB216763, supports this pathway as a promising target for pharmacological intervention in FGR.

## Data Availability

The original contributions presented in the study are included in the article/Supplementary Material, further inquiries can be directed to the corresponding authors.

## Abbreviations

AGA, Appropriate for Gestational Age; ANOVA, Analysis Of Variance; Bmp, Bone Morphogenetic Protein; CDD, Charge-Coupled Device; CREB, cAMP response Element-Binding protein; EGFP, enhanced Green Fluorescent protein; FGF, Fibroblast Growth Factor; FGR, Foetal Growth Restriction; GAPDH, GlycerAldehyde 3-Phosphate DeHydrogenase; Hif, Hypoxia Inducible Factor; IGF, Insulin-like Growth Factor; IGFBP1, Insulin-like Growth Factor Binding Protein one; IUGR, Intrauterine Growth Restriction; Jak/Stat, Janus kinase/Signal Transducer and Activator of Transcription protein; OPBA, Organismo Preposto al Benessere degli Animali; qPCR, quantitative real-time Polymerase Chain Reaction; ROS, Reactive Oxygen Species; Shh, Sonic-Hedgehog; TGFβ, Transforming Growth Factor Beta; WISH, Whole-mount *In Situ* Hybridization; Wnt, Wingless/Int1; YAP-TAZ, Yes-Associated Protein-Transcriptional coActivator with PDZ-binding motif; ZIRC, Zebrafish International Resource Centre; Declarations.
