# Supplementary material for: Hyperactivation of Wnt/β-catenin and Jak/Stat3 pathways in human and zebrafish foetal growth restriction models: Implications for pharmacological rescue
Source: Front Cell Dev Biol. 2022 Aug 16;10:943127. doi: 10.3389/fcell.2022.943127 (PMC9424487; doi:10.3389/fcell.2022.943127)
Supplement: Supplementary file 1 [file DataSheet1.pdf]

Supplementary data

Supplementary Table 1: PCR primers and conditions for gene expression analysis by quantitative real-time PCR

Gene name	Signalling pathway	Forward primer (5'-3')	Reverse primer (5'-3')	Product size (bp)
Human analysis				
GAPDH	Housekeeping	GGATTTGGTCGTATTGGG	GGAAGATGGTGATGGGATT	205
IGFBP1	IGF	AGGCACAGGAGACATCAGGA	CCATTCCAAGGGTAGACGCA	142
IGFPB7	IGF	TCCAATTCCCAAGGACAGGC	TATAGCTCGGCACCTTCACC	98
CCND1	Wnt/β-catenin	GATGCCAACCTCCTCAACGAC	CTCCTCGCACTTCTGTTCCTC	171
MYC	Wnt/β-catenin	TTCTCTCCGTCCTCGGATTC	GTAGTTGTGCTGATGTGTGGA	282
FST	Wnt/β-catenin	CAATGCCACTTATGCCAGCG	GCTCAGGTTTTACGGGCAGA	115
CTNNB1	Wnt/β-catenin	TTCGCCTTCACTATGGACTACC	GCACGAACAAGCAACTGAACTA	175
VEGFA	Hif/Hypoxia	AGGCCAGCACATAGGAGAGA	TACCGGGATTTCTTGCGCTT	141
ID1	CREB	CAGGGACCTTCAGTTGGAGC	CTTCAGCGACACAAGATGCG	165
ID3	BMP	GCTCACTCCGGAACTTGTCA	TGGTGAAGTCAAGTGGGCAG	185
SMO	Shh	CCTGCTCACCTGGTCACTC	CACGGTATCGGTAGTTCTTGTAG	119
SMAD2	TGFβ	ACCGAAATGCCACGGTAGAA	TGGGGCTCTGCACAAAGAT	123
SMAD3	TGFβ	CATCGAGCCCCAGAGCAATA	GTGGTTCATCTGGTGGTCACT	88
TGFB1	TGFβ	CCCCTACATTTGGAGCCTGG	GCACGATCATGTTGGACAGC	176
NOTCH1	Notch	TGCGAGACCAACATCAACGA	AGGTTGATCTCGCAGTTGGG	128
HES1	Notch	CTACCCCAGCCAGTGTCAAC	GTCCGCCTTCTCCAGCTTG	191
JAG1	Notch	AATGGCTACCGGTGTGTCTG	CCCATGGTGATGCAAGGTCT	83
CCN2 (CTGF)	Hippo/YAP-TAZ	CTTGCGAAGCTGACCTGGAA	AAAGCTCAAACTTGATAGGCTTGGA	90
DUSP6	FGF	TCTACGACGAGAGCAGCAG	GGAGAACTCGGCTTGGAACT	143
SERPINE1	Jak/Stat3	TCTGCCCTCACCAACATTCT	CGGTCATTCCCAGGTTCTCT	148
NR3C1	Glucocorticoid	GAAGGAAACTCCAGCCAGAA	CAGCTAACATCTCGGGGAAT	151
EDN1	Oestrogen	TGTGTCTACTTCTGCCACCT	TTCACGGTCTGTTGCCTTTG	132
EDNRB	Oestrogen	TCTCTGTGGTTCTGGCTGTC	AGCCACCAATCTTTTGCTGT	148
Zebrafish analysis				
gapdh	Housekeeping	GTGGAGTCTACTGGTGTCTTC	GTGCAGGAGGCATTGCTTACA	173
igfbp1a	IGF	GAACTCCAGACAGCCCTTGA	CAGGATGACACACACCAAC	167
igfbp1b	IGF	GGCACAGGAGAGCATCAAGT	GGGCAGGTAGAAACTGGTGA	151

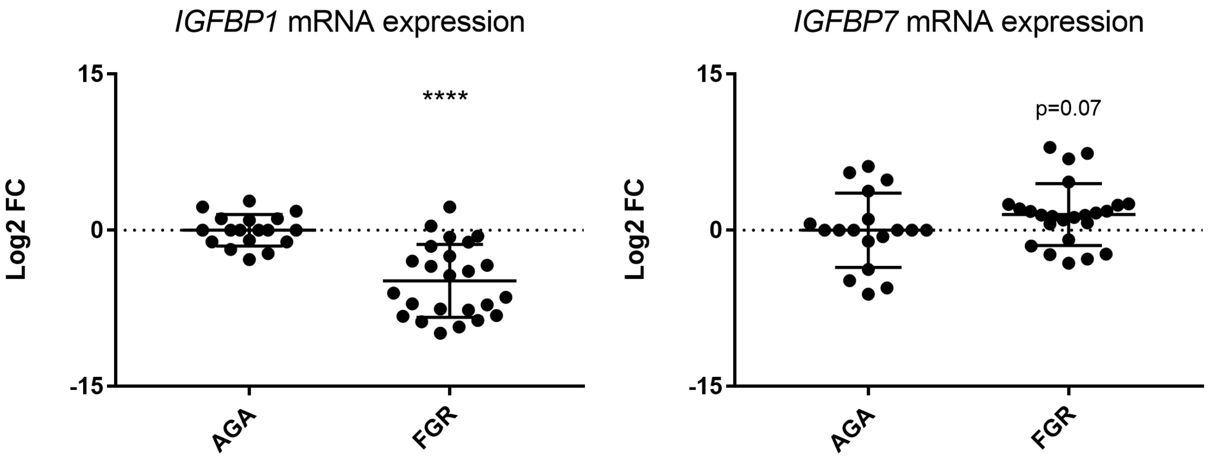

Supplementary Figure 1: Dysregulation of the IGF pathway in human FGR cases.

Quantitative RT-PCR analysis, performed on umbilical cords from FGR cases and AGA controls, focusing on IGF signalling members, shows significant reduction of *IGFBP1* and a slight ($p=0.07$) increase of *IGFBP7*. Sample size: $n=24$; ****=$p<0.0001$; Log2 FC stands for Log2 Fold Change. Test: Unpaired t-test.

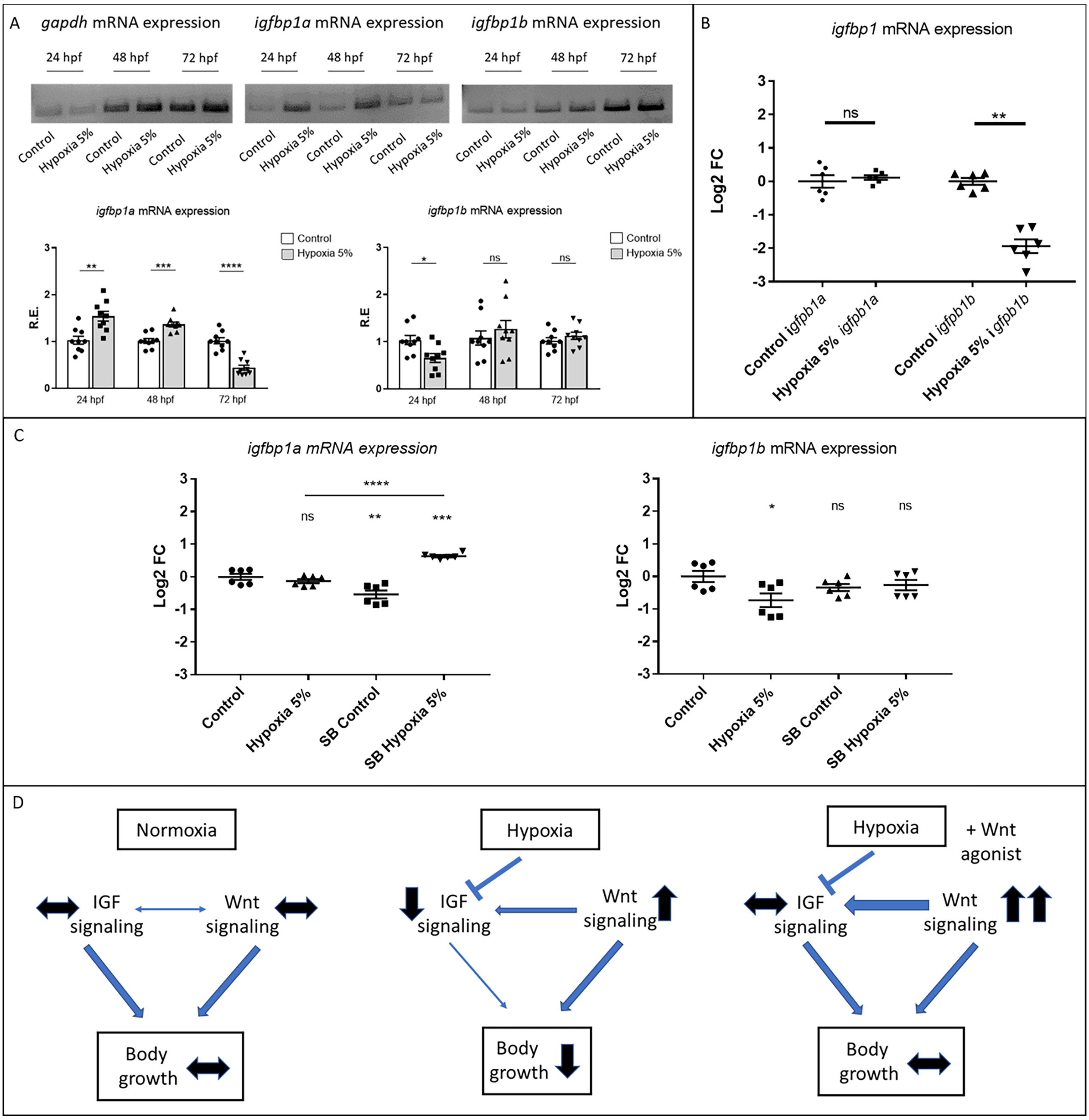

Supplementary Figure 2: Modification of zebrafish *igfbp* gene expression under short- and long-term hypoxia

A: Treatment of zebrafish embryos with 1-day long incubations in 5% hypoxia induces gene expression changes in IGF signalling members *igfbp1a* and *igfbp1b* when analysed at 24, 48 and 72 hpf, in comparison with normoxia controls. Data are normalized to the housekeeping gene *gapdh*. Sample size: n=30. R.E.=mRNA Relative Expression.

B: Treatment of zebrafish embryos with continuous (2-day long) 5% hypoxia results in the downregulation of the IGF signalling member *igfbp1b*, while the paralog *igfbp1a* remains substantially unaffected, compared to normoxia controls, when analysed at 72 hpf. Data are normalized to the housekeeping gene *gapdh*. Sample size: n=30; ns=not significant; **=p<0.01. Log2 FC stands for Log2 Fold Change. Test: Unpaired t-test. C: Analysis of *igfbp1a* and *igfbp1b* genes under 5% hypoxia confirm their expressional behaviour (stability and downregulation, respectively), while treatment with the Wnt agonist SB216763 (SB), at 40 µM, under 5% hypoxia, leads to *igfbp1a* upregulation and *igfbp1b* rescue, compared to untreated controls under 5% hypoxia. Sample size: n=15; ns=not significant; *=p<0.05; **=p<0.01; ***=p<0.001; ****=p<0.0001. Log2 FC stands for Log2 Fold Change. Test: One-way ANOVA followed by Tukey's test. D: Model of IGF/Wnt signalling interaction under normoxia and 5% hypoxia. Under normoxia, balanced levels of IGF and Wnt signalling ensure a physiological body growth. Under hypoxia, IGF signalling is downregulated and the body growth is reduced; physiological upregulation of Wnt signalling is unable to fully rescue the body size. Under hypoxia and chemical hyper-activation of Wnt signalling, IGF signalling is rescued/upregulated and the body growth is restored to normal levels.

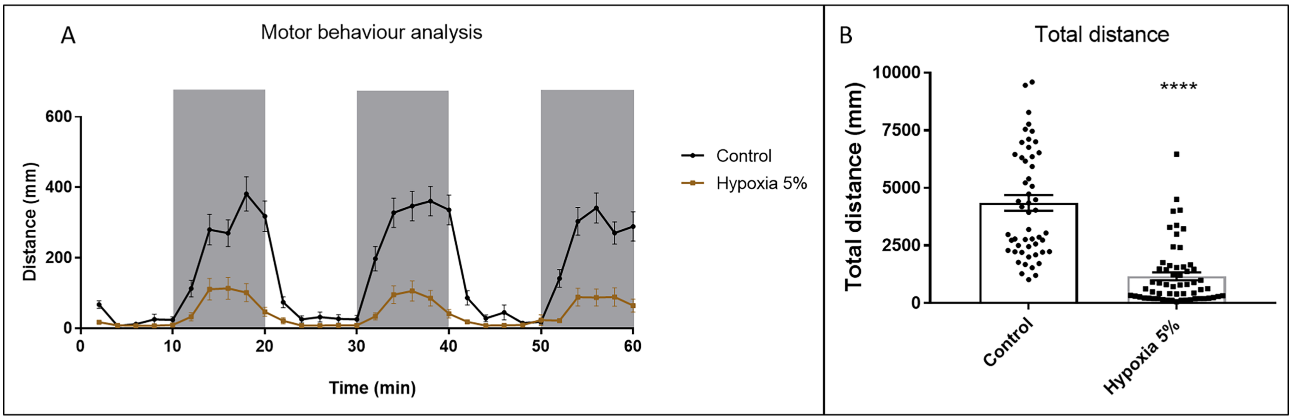

Supplementary Figure 3: Impaired motor behaviour in hypoxia-treated zebrafish

(A) Wild type larvae at 4 dpf, either untreated (black line) or 5% hypoxia-treated for 2 days (orange line) display normal response to light (white areas) and dark (grey areas) stimuli (A), but with reduced motor performances, evaluated as total distance swum (B). Sample size: n=60; ****=p<0.0001. Test: Unpaired t-test.

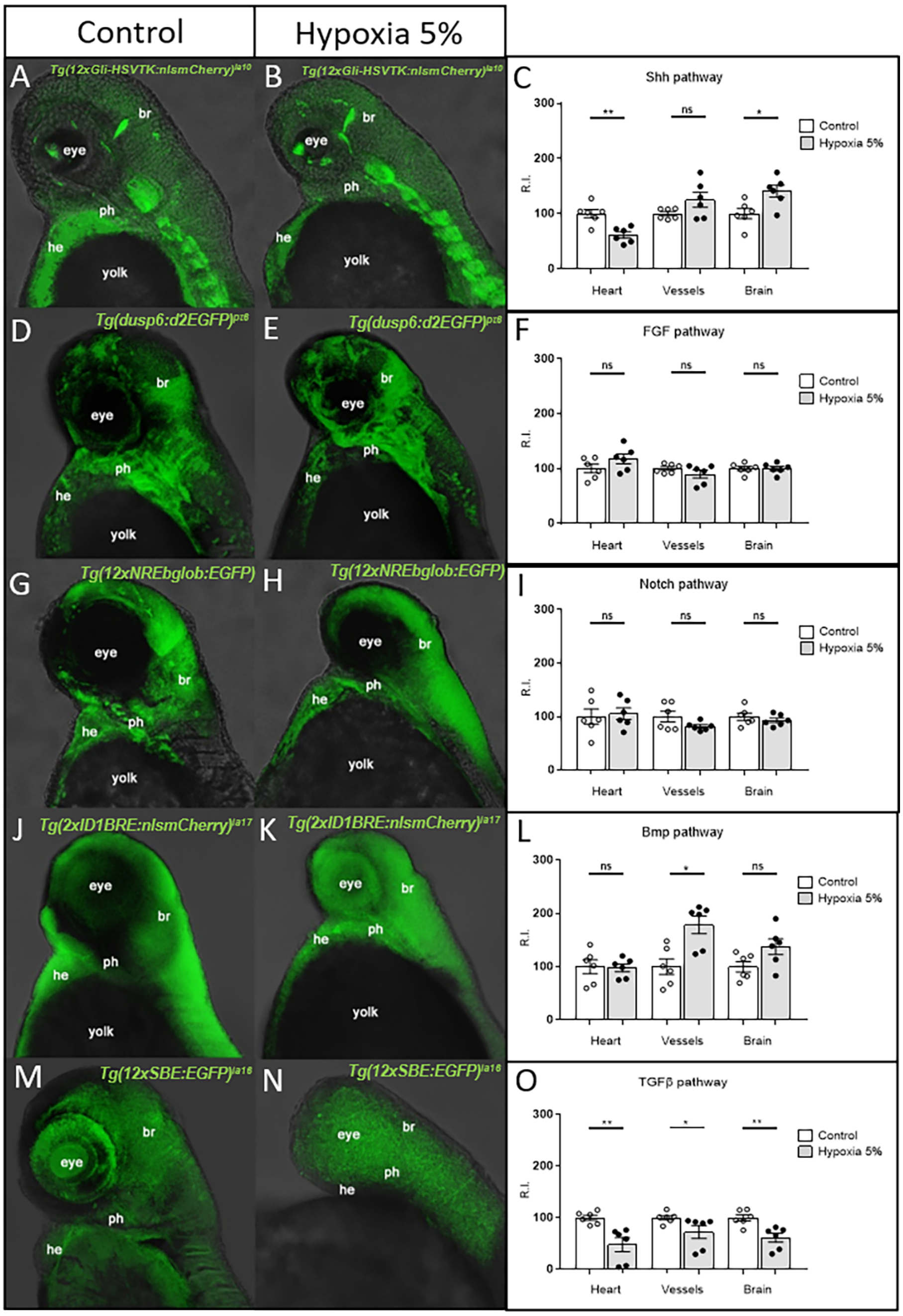

Supplementary Figure 4: Multiple pathway analysis in hypoxia-treated zebrafish embryos

The GFP-based (green) reporters show unmodified or mildly affected regulation of the following pathways in the cardiovascular and brain regions of hypoxia-treated embryos: Shh (A-D), FGF (D-F), Notch (G-I), and Bmp (J-L). TGFβ signalling (M-O) is downregulated in all considered regions. All embryos are at 3 dpf and displayed in lateral view, anterior to the top; br=brain; he=heart; ph=pharynx. Sample size: n=6 per condition; ns=not significant; *=p<0.05; **=p<0.01; R.I.=Relative Intensity. Test: Unpaired t-test.

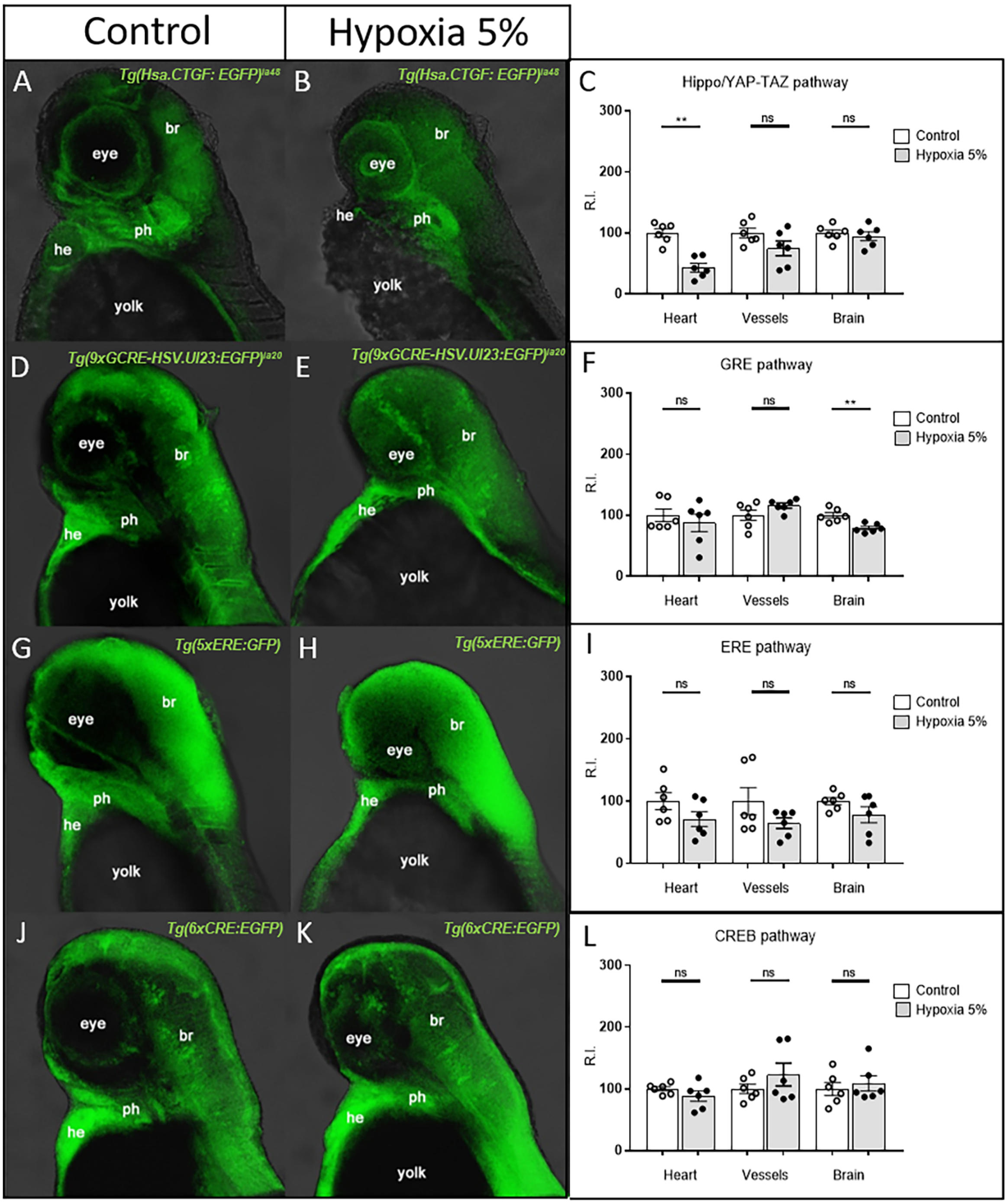

Supplementary Figure 5: Multiple pathway analysis in hypoxia-treated zebrafish embryos

The GFP-based (green) reporters show unmodified or mildly affected regulation of the following pathways in the cardiovascular and brain regions of hypoxia-treated embryos: Hippo/YAP-TAZ (A-C), GRE (D-F), ERE (G-I) and CREB (J-L). All embryos are at 3 dpf and displayed in lateral view, anterior to the top; br=brain; he=heart; ph=pharynx. Sample size: n=6 per condition; ns=not significant; **=$p<0.01$; R.I.=Relative Intensity. Test: Unpaired t-test.
